# Expanding the Current Knowledge About the Role of Interleukin-10 to Major Concerning Bacteria

**DOI:** 10.3389/fmicb.2018.02047

**Published:** 2018-09-18

**Authors:** Hernán F. Peñaloza, Loreani P. Noguera, Claudia A. Riedel, Susan M. Bueno

**Affiliations:** ^1^Millennium Institute on Immunology and Immunotherapy, Departamento de Genética Molecular y Microbiología, Facultad de Ciencias Biológicas, Pontificia Universidad Católica de Chile, Santiago, Chile; ^2^Millennium Institute on Immunology and Immunotherapy, Departamento de Ciencias Biológicas, Facultad de Ciencias de la Vida, Universidad Andrés Bello, Santiago, Chile

**Keywords:** interleukin-10, multi-drug resistance, bacterial infection, immune response, host–pathogen interactions

## Abstract

Interleukin-10 (IL-10) is one of the most important anti-inflammatory cytokine produced during bacterial infection. Two related phenomena explain the importance of IL-10 production in this context: first, the wide range of cells able to produce this cytokine and second, the wide effects that it causes on target cells. In a previous report we described opposing roles of IL-10 production during bacterial infection. Overall, during infections caused by intracellular bacteria or by pathogens that modulate the inflammatory response, IL-10 production facilitates bacterial persistence and dissemination within the host. Whereas during infections caused by extracellular or highly inflammatory bacteria, IL-10 production reduces host tissue damage and facilitates host survival. Given that these data were obtained using antibiotic susceptible bacteria, the potential application of these studies to multi-drug resistant (MDR) bacteria needs to be evaluated. MDR bacteria can become by 2050 a major death cause worldwide, not only for its ability to resist antimicrobial therapy but also because the virulence of these strains is different as compared to antibiotic susceptible strains. Therefore, it is important to understand the interaction of MDR-bacteria with the immune system during infection. This review discusses the current data about the role of IL-10 during infections caused by major circulating antibiotic resistant bacteria. We conclude that the production of IL-10 improves host survival during infections caused by extracellular or highly inflammatory bacteria, however, it is detrimental during infections caused by intracellular bacteria or bacterial pathogens that modulate the inflammatory response. Importantly, during MDR-bacterial infections a differential IL-10 production has been described, compared to non-MDR bacteria, which might be due to virulence factors specific of MDR bacteria that modulate production of IL-10. This knowledge is important for the development of new therapies against infections caused by these bacteria, where antibiotics effectiveness is dramatically decreasing.

## Introduction

Infectious diseases caused by pathogenic bacteria have been historically a major cause of human death. The discovery and mass production of antibiotics dramatically reduced the mortality associated to bacterial infections for several years. However, the golden age of antibiotic seems to be over: according to a report led by the economist Jim O’Neill, in 2017 around 700,000 deaths were caused by multi-drug resistant (MDR) bacterial infections. His prediction, however, indicates that by 2,050 the mortality associated to MDR-bacterial infections will be around of 10 million (O’Neill, 2016).

Due to this phenomenon, the World Health Organization (WHO) has classified the most concerning MDR-bacteria into different groups of priority according with the urgency to develop new antibiotics and therapies (Tacconelli, 2017). The critical priority group includes *Acinetobacter baumannii*, *Pseudomonas aeruginosa*, and *Mycobacterium tuberculosis.* Also, different species of the *Enterobacteriaceae* genus, including *Klebsiella pneumoniae*, *Escherichia coli*, are part of this group. The high priority group includes the pathogenic bacteria *Staphylococcus aureus*, *Neisseria gonorrhoeae*, *Salmonella* spp., among others.

In a previous report we discussed the relevance of the anti-inflammatory cytokine Interleukin-10 (IL-10) in the immunity against different pathogenic bacteria (Peñaloza et al., 2016). We conclude that IL-10 production is required for host survival during infections caused by extracellular and/or highly pro-inflammatory bacteria, including *Streptococcus pneumoniae*, *Pseudomonas aeruginosa*, *Francisella tularensis*, *Escherichia coli*, and *Mycobacterium tuberculosis* (Peñaloza et al., 2016). On the other hand, IL-10 production impairs host survival during infections caused by intracellular bacteria or bacterial pathogens that modulate the inflammatory response, such as *Klebsiella pneumoniae*, *Bordetella pertussis*, *Listeria monocytogenes*, *Brucella abortus* and *Salmonella enterica* serovar Typhimurium (Peñaloza et al., 2016).

During extracellular or/and highly pro-inflammatory bacterial infection, the pathogen mostly neutralizes or eliminates the immune response effectors. In this context, the production of IL-10 modulates the immune response intensity and allows a successful bacterial clearance, without excessive host tissue damage. Although in some cases the absence of IL-10 makes the immune response more effective to clear the pathogen, the damage produced on host tissues is more severe and compromise host survival (Peñaloza et al., 2016).

The strategies used by intracellular pathogens and/or bacteria that modulate the inflammatory response are different and mainly consist in their capacity to evade the immune system (Bhavsar et al., 2007). In this condition, the production of IL-10 during the infective cycle of these bacteria helps them to evade the immune response and to disseminate within the host, seriously impairing host survival.

The studies performed so far that evaluate the role of IL-10 during bacterial infections have been performed using antibiotic susceptible bacteria. The conclusions reached in these reports cannot be translated to MDR-bacteria. Recent data have described that the immune response against antibiotic susceptible and MDR strains of the same bacterial specie may be remarkably different (de Breij et al., 2012; Xiong et al., 2015). Therefore, it raises two major questions about infections caused by MDR-bacteria. First: is the available data accurate enough to predict the role of immune effectors, such as anti-inflammatory cytokines, during MDR bacterial infections? And second: is it possible to develop new therapies against MDR bacteria with the current knowledge? To date, there are not enough data available to certainly answer both of these questions. However, in the last years increased efforts have made possible to understand better the role of some immune components in the response against different MDR bacteria.

In this mini-review, we will discuss whether the role of IL-10 during infections caused by major antibiotic susceptible bacteria applies to major MDR-bacterial infections. Next, we will analyze whether MDR-bacteria induce a differential production of IL-10 as compared to non-MDR bacteria, to finally discuss about the possible mechanisms that may explain this difference.

## The Opposing Role of Il-10 During Multi-Drug Resistant Bacterial Infection

Carbapenem-resistant *Acinetobacter baumannii* (*A. baumannii*) is an opportunistic, aerobic, Gram-negative, and extracellular bacteria classified as a critical pathogen by the WHO (Tacconelli, 2017). *A. baumannii* is able to evade neutrophil mediated killing, including neutrophil extracellular traps (NETs) degradation (Kamoshida et al., 2015) or uptake inhibition after adherence (Kamoshida et al., 2015). This bacterium is commonly associated to primary health care facilities, causing skin infections, pneumonia, urinary tract infections and sepsis, mostly in immunocompromised patients (Lee et al., 2017; Harding et al., 2018).

One study demonstrated that during *A. baumannii* pneumonia, MDR *A. baumannii* virulence varies significantly as compared to non-MDR-*A. baumannii* (de Breij et al., 2012). Importantly, the production of IL-10 in the lungs was positively correlated with host survival (de Breij et al., 2012). Another report supported this observation (Noto et al., 2017). That report evaluated the role of receptor for advanced glycation end products (RAGE) during an *A. baumannii* infection. RAGE^-/-^ mice are more resistant to a pneumonia caused by non-MDR *A. baumannii* (Noto et al., 2017). The elevated survival rate displayed by RAGE^-/-^ mice is dependent on IL-10. Given that the neutralization of this cytokine reversed the resistant phenotype observed in these mice, and at the same time the administration of recombinant IL-10 improves host survival of susceptible wild type (WT) mice (Noto et al., 2017).

Carbapenem-resistant *Pseudomonas aeruginosa* (*P. aeruginosa*) is another bacterium classified in the critical group (Tacconelli, 2017). *P. aeruginosa* is a Gram-negative opportunistic pathogenic bacterium able to causes chronic respiratory tract infections in patients with cystic fibrosis and in patients with compromised immune system (Gellatly and Hancock, 2013). Due to the importance of *P. aeruginosa*, since long time ago scientists have started to use clinical isolates to study host-pathogen interaction, specifically ampicillin resistant strains (Chmiel et al., 1999, 2002; Parker et al., 2016; Riquelme et al., 2017). A recent study analyzed cytokine production, cellular recruitment and bacterial clearance during the first 12 h during an intraperitoneal infection with four MDR isolates, three non-MDR isolates and two reference strains of *P. aeruginosa* in mice (Gomez-Zorrilla et al., 2017a). MDR isolates induced a stronger immune response able to clear more efficiently the infective bacteria at 12 hpi (Gomez-Zorrilla et al., 2017a). These data help to understand the differences of virulence and pathogenicity between MDR and not-MDR strains. However, given that the infection route was intraperitoneally and not intranasal, we cannot conclude whether the production of IL-10 is beneficial or detrimental during pneumonia caused by MDR *P. aeruginosa* in this particular experiment. Despite that, all the evidences show that IL-10 production protects the host during *P. aeruginosa*.

Both Carbapenem-resistant and 3^rd^ generation of cephalosporin resistant *Enterobacteriaceae* have also been categorized in the critical group (Tacconelli, 2017). Within this group are included *Klebsiella pneumonia*, *Escherichia coli*, *Enterobacter* spp., *Serratia* spp., *Proteus* spp., *Providencia* spp. and *Morganella* spp. Most of the studies performed on these bacteria are epidemiological (Liñares et al., 2010; Rojas et al., 2017), genetic (Deleo et al., 2014; Rojas et al., 2017), or have been focused to understand the mechanisms involved in the acquisition of antibiotic resistance (Fuzi et al., 2017; Dhar et al., 2018). However, few studies have been focused in the analysis of host-pathogen interaction with MDR-strains. Regarding to that these reports describe the immune response against carbapenem-resistant *K. pneumoniae* (CRKP) (Tzouvelekis et al., 2013; Xiong et al., 2015; Ahn et al., 2016). These studies showed that CRKP isolates are less virulent (Tzouvelekis et al., 2013; Xiong et al., 2015) than antibiotic susceptible *K. pneumoniae* strains, but resistant to neutrophil-mediated clearance (Tzouvelekis et al., 2013; Ahn et al., 2016; Kobayashi et al., 2016). CRKP showed to be highly susceptible to human serum (Olonisakin et al., 2016; DeLeo et al., 2017) and the anti-capsule antibodies generation helped to innate cells to clear this pathogen *in vivo* (Kobayashi et al., 2018). Another important characteristic of CRKP is the primary role of monocytes in the pathogenesis caused by this bacterium. For monocytes, at least two different roles have been described during CRKP infection: some of the monocytes recruited to the lungs are Monocytic Myeloid-derived cells (M-MDSCs) (Ahn et al., 2016) probably able to establish an anti-inflammatory environment, aiding CRKP to evade the immune response. On the other hand, inflammatory monocytes are essential to phagocyte and kill CRKP as well as to activate ILC3s and Th_17_ response (Xiong et al., 2016).

*Mycobacterium tuberculosis* (*M. tuberculosis*) is another bacterium included in the critical WHO group. *M. tuberculosis* is a rod-shaped bacterium with a cell wall rich in lipids able to cause chronic and latent infections in the lung tissue (Glickman and Jacobs, 2001; Bañuls et al., 2015). During non-MDR *M. tuberculosis* infections, IL-10 production by neutrophils is important for host survival (Peñaloza et al., 2016). But there is not data available that describe the immunity and the role of IL-10 during MDR *M. tuberculosis* infection at molecular level.

Methicillin/Vancomycin-resistant *Staphylococcus aureus* and 3rd generation cephalosporin/fluoroquinolone-resistant *Neisseria gonorrhoeae (N. gonorrhoeae)* are the only WHO high priority bacterial species for which the role of IL-10 production has been described (Liu et al., 2013; Leech et al., 2017). *Staphylococcus aureus* is an extracellular Gram-positive bacterium able to cause serious lung (Kitur et al., 2015) and skin infections (Kitur et al., 2016). Most of host-pathogen interaction research has been done on Methicillin-resistant *S. aureus* (MRSA), and the result of this research is the identification of bacterial and host factors involved in MRSA infection (Parker et al., 2016; Leech et al., 2017).

During a MRSA (USA300) infection there is a rapid production of IL-10 (Leech et al., 2017). The contribution of this cytokine for host survival varies according the nature of the infection. During an acute systemic infection, the production of IL-10 by CD19^+^CDB220^+^ cells was required to control the bacterial dissemination and its production is associated with host survival (Leech et al., 2017). However, in a sub-cutaneous infection model, IL-10 produced mostly by MDSCs in the local wound impairs MRSA clearance, without affecting host survival (Leech et al., 2017). MRSA infects orthopedic devices. In this case, the production of IL-10 by MDSCs favors the biofilm formation on the surface of these devices inducing persistent infections that do not affect host mortality (Heim et al., 2014, 2015).

*Neisseria gonorrhoeae* is an obligate Gram-negative human pathogen (Balthazar et al., 2011; Araneta et al., 2017) causative of gonorrhea, a sexually transmitted disease (Balthazar et al., 2011; Araneta et al., 2017). During an intra-vaginal *N. gonorrhoeae* infection, the neutralization of IL-10 through the administration of microspheres improved the clearance of *N. gonorrhoeae* and enhanced the polarization to a Th_1_ response (Liu et al., 2013). These findings suggest that the production of IL-10 is detrimental for the immunity against this bacterium (Liu et al., 2013).

Fluoroquinolone-resistant *Salmonella enterica* (*S. enterica)* is a pathogenic bacterium categorized in the high priority group with *S. aureus* and *N. gonerrhoeae* (Tacconelli, 2017). *S. enterica* is a Gram-negative foodborne pathogenic bacterium (Lamas et al., 2018). Similar to other bacteria, few host-pathogen interaction studies have been reported for MDR *Salmonella* and yet the role of IL-10 during MDR *Salmonella* infection remains unknown. However, we have recently reported the role of IL-10 during an infection with non-MDR-*Salmonella enterica* serovar Typhimurium (Salazar et al., 2017). This report demonstrates that IL-10 produced mostly by T cells impairs *Salmonella* clearance and increases host mortality (Salazar et al., 2017). Further research is needed to evaluate whether MDR-*S*. Typhimurium will differentially induce IL-10 production during infection.

## Mdr and Non-Mdr Bacteria Induce Differential Il-10 Production During Infection

In the previous section, we described that the production of IL-10 plays a critical role during MDR-bacterial infections. Similar to infections caused by non-MDR bacteria, the production of IL-10 may lead to a better or worse outcome depending of the infecting bacteria. Since the MDR-bacteria and non-MDR bacteria present different virulence, it is possible to hypothesize that MDR-bacteria induce differential amounts of IL-10 during infection. Indeed, several studies corroborate this hypothesis. For example, ICU patients infected with extensively drug-resistant (XDR) *P. aeruginosa* presented elevated amounts of IL-10 in blood, as compared to patients infected with non-MDR *P. aeruginosa* (Gomez-Zorrilla et al., 2017b). Same result was obtained in a mouse model of *P. aeruginosa* pneumonia (Tam et al., 2018). Consistently, patients infected with MDR *M. tuberculosis* presented higher amounts of IL-10 in the serum, as compared to patients infected with non-MDR *M. tuberculosis* (Pinheiro et al., 2013). However, another group did not find any difference in IL-10 production in patients infected with MDR or non-MDR *M. tuberculosis* (Eum et al., 2008). Moreover, during a *S. aureus* endocarditis model, rabbits infected with MRSA presented higher amounts of IL-10 as compared to those infected with Methicillin susceptible *S. aureus* in the serum (Tsaganos et al., 2013).

All these data suggest that MDR-bacteria might induce more IL-10 production than non-MDR bacteria. The only exception was found in *A. baumannii* infection, where children infected either with MDR *A. baumannii* presented the same levels of IL-10 as compared with children infected with non-MDR *A. baumannii* (Fang et al., 2016).

**FIGURE 1:**
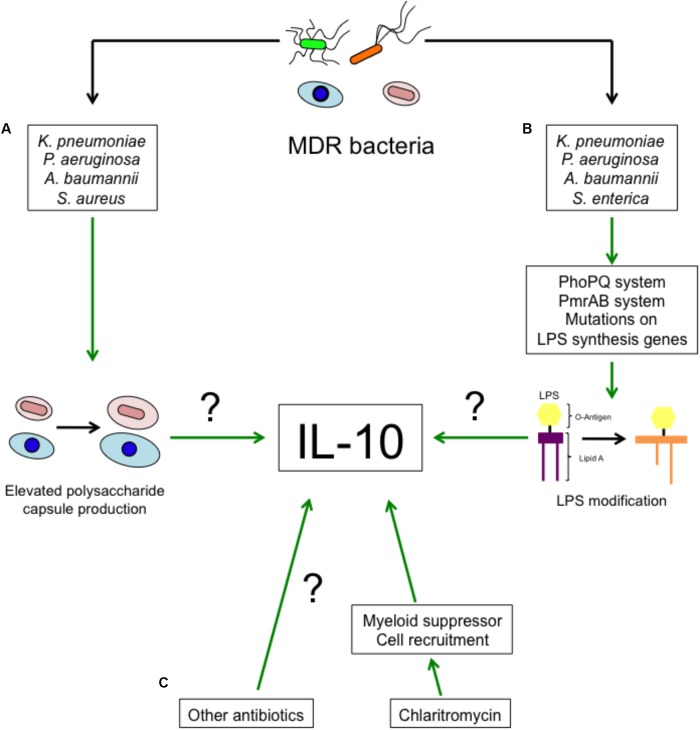
Proposed mechanisms of enhanced IL-10 production during MDR-bacterial infections and antibiotic treatment. The increased IL-10 production during MDR-bacteria can possibly be a consequence of **(A)** an improved ability of bacteria to synthesize polysaccharide capsule or **(B)** modifications in LPS. **(C)** Clarithromycin and probably other antibiotics promote the recruitment of myeloid suppressor cells and IL-10 production.

The next question then is as to how MDR-bacteria induce higher levels of IL-10. It is well known that antibiotic resistance not only involves the acquisition of hydrolytic enzymes, but also includes changes in proteins structure or expression of genes involved in virulence such as porins, two-component systems and others (Beceiro et al., 2013). Polysaccharide capsule (**Figure 1A**), present in bacteria such as *K. pneumoniae, P. aeruginosa, A. baumannii, S. aureus*, among other bacteria, is an important determinant for antimicrobial peptides and Polymyxin B resistance (Llobet et al., 2008). An increased ability to produce capsule may be an adaptation against antibiotics. Interestingly, capsule recognition by pattern recognition receptors (PRRs) triggers IL-10 production in myeloid cells (Peñaloza et al., 2016). MgrB is an important regulator of gene expression in *K. pneumoniae*, deletion of *mgrB* increases the resistance to collistin and Polymyxin B by the remodeling of lipid A mediated by the PhoPQ system (Kidd et al., 2017). This increased resistance is associated with increased virulence and reduced TNF-α production (Kidd et al., 2017). Lipid A remodeling has also been observed in MDR *P. aeruginosa*, also dependent on the activity of the PhoPQ system (Barrow and Kwon, 2009; Lee et al., 2017). PmrAB, another two-component system, is also involved in LPS modification and promotes antibiotic resistance in *S. enterica* and *P. aeruginosa* (Gunn et al., 2000; Barrow and Kwon, 2009). Similarly, *A. baumannii* resistance to Polymyxin B is mediated by mutations in *lpxA, lpxC* and *lpxD*. These mutations lead to the loss of LPS by altering the biosynthesis of the lipid A (Moffat et al., 2010) and induce a weaker immune response. It is unknown whether LPS modification by MDR-bacteria leads to higher production of IL-10. This possibility is highly probable, given that LPS recognition by TLR4 induces IL-10 production in myeloid cells (**Figure 1B**) (Peñaloza et al., 2016).

Multi-drug resistant *N. gonorrhoeae* carries the *opa* gene (Hess et al., 2012). Opa inhibits the proliferation of CD4^+^ T cells and B cells in mice (Boulton and Gray-Owen, 2002), increases the expression of PD-L1 and stimulates the production of IL-10 (Zhu et al., 2012).

Even though the treatments against MDR bacteria are limited, they mostly consist of antibiotic administration. A recent report describe that Clarithromycin promotes the recruitment of anti-inflammatory myeloid cells able to produce IL-10, Arginase-1 and iNOS to the lung tissue and the spleen (Namkoong et al., 2018). The recruitment of these cells to the lung protects the host during pneumococcal pneumonia and in LPS endotoxin shock (Namkoong et al., 2018). It is unknown whether other antibiotics also induce the production of IL-10 or other anti-inflammatory mediators. This would be an interesting hypothesis to evaluate (**Figure 1C**).

## Conclusion

In the last years, increasing data support the idea that IL-10 production drives the development of a successful immune response during bacterial infection. However, whether IL-10 production is beneficial or not for host survival depends of the pathogen nature and the immune response associated to the infection (Peñaloza et al., 2016).

The rapid emergence of MDR bacteria is a major concern and has pushed the research community to invest more efforts in studying these bacteria. Most of the research done on MDR bacteria has been focused on epidemiological and genetic characterization (Liñares et al., 2010; Deleo et al., 2014; Rojas et al., 2017); and limited numbers of studies have been focused in the understanding of host-pathogen interaction. The most important reason for the existence of this gap is the high amount of data available regarding non-MDR strains and the assumption that virulence and pathogenicity of MDR and non-MDR bacteria are similar. Recent reports show that pathogenicity and virulence can be remarkably different between MDR and non-MDR strains (de Breij et al., 2012; Tzouvelekis et al., 2013; Xiong et al., 2015).

The data discussed in this mini-review support the dual role of IL-10, previously described for non-MDR can also be found during MDR bacterial infection. For non-MDR bacterial infection IL-10 production is required for host survival during infections caused by extracellular and/or highly pro-inflammatory bacteria. Conversely, for MDR bacterial infection, IL-10 impairs host survival and bacterial clearance during intracellular and/or weak pro-inflammatory bacteria. During *A. baumannii* and MRSA infection, the production of IL-10 is required for host survival. Both bacteria have acquired different virulence factors that allow them to neutralize the host immune response at different stages. In this scenario, a hyperactive immune response may occur, being IL-10 essential to modulate the intensity of it (**Figure 1**, left panel).

As we discussed previously, most of the studies about host–pathogen interaction of *P. aeruginosa* has been done using ampicillin resistant strains. In this scenario the production of IL-10 seems to be required for host survival, however, whether these data can be translated to carbapenem-resistant *P. aeruginosa* is unknown and more studies need to be done.

**FIGURE 2:**
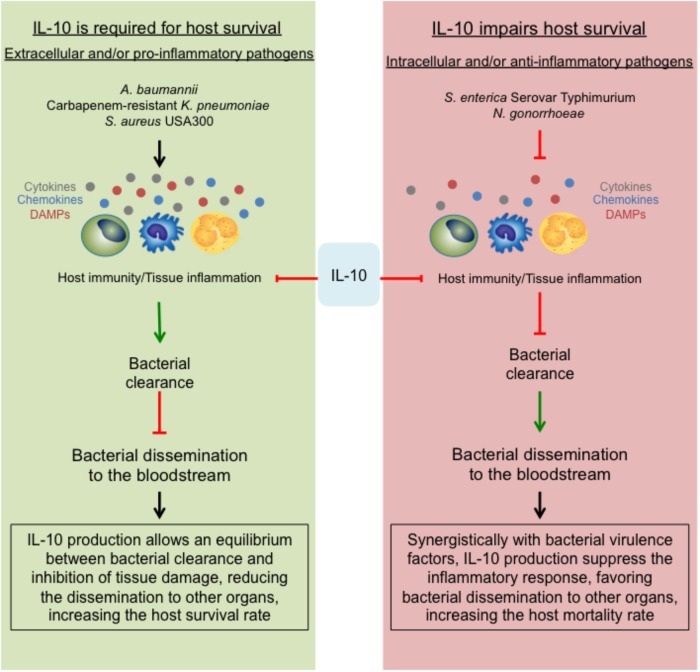
The opposing role of the anti-inflammatory effect of IL-10. During MDR bacterial infections, the suppressive role of IL-10 can lead into two different outcomes. **(Left)** Describes that during infections with extracellular and/or pro-inflammatory bacteria, the strong immune response will be essentially modulated by IL-10, allowing a slow clearance with limited host tissue damage, generating an equilibrium required for host survival. On the contrary, during infections with intracellular and/or anti-inflammatory bacteria **(right)**, the production of IL-10 together with the bacterial virulence strategies will shut down the immune response, impairing the bacterial clearance, facilitating the dissemination within the host and at the end, increasing the host mortality rate.

*S. enterica* and *N. gonorrhoeae* are both intracellular pathogens, either way; IL-10 absence and/or neutralization improve the host immunity against these microbes and increase host survival (**Figure 2**, right panel). Overall, these data is consistent with our initial hypothesis.

Carbapenem-resistant *K. pneumoniae* is a special case. *K. pneumoniae* is an extracellular bacterium that induces a weak pro-inflammatory response (Ahn et al., 2016). Published data show that during non-MDR *K. pneumoniae* infection, IL-10 production facilitates bacterial dissemination and impairs host survival (Peñaloza et al., 2016). Several reports show that the virulence and pathogenicity of MDR strains are reduced as compared to non-MDR strains (Tzouvelekis et al., 2013; Xiong et al., 2015; Ahn et al., 2016). It is quite remarkable that even though CRKP doses typically used in mice studies are around 1 × 10^8^ CFUs/mice, WT C57BL/6J are highly resistant to these infections (Tzouvelekis et al., 2013; Xiong et al., 2015; Ahn et al., 2016). These bacteria are also resistant to neutrophil-mediated clearance (Ahn et al., 2016; Kobayashi et al., 2016) and during CRKP there is a rapid peak of IL-10 production (Ahn et al., 2016). Taking account all this data, we hypothesize that during CRKP infection IL-10 is a major immune response modulator required for host survival (**Figure 2**, left panel).

One of the most important concerns of this report is the lack of research regarding host–pathogen interaction on MDR bacteria. It is clear by the whole community that the golden age of antibiotics is over, however, the knowledge of how these bacteria have adapted to the host immune response and how we can improve it is very limited. Therefore, we believe that more research in host immunity and bacterial virulence is required to design new strategies to identify effective therapeutic targets or strategies.

Finally, it is quite interesting MDR-bacteria stimulates higher IL-10 production as compared with non-MDR bacteria. We believe that the differential production of IL-10 may be a consequence of the changed expression pattern of different virulence factors displayed by these bacteria. This fact could explain why in general these bacteria are less virulent and why higher bacterial dose are commonly used to study host–pathogen interaction in mice.

## Author Contributions

All authors listed have made substantial, direct, and intellectual contribution to the work and approved it for publication.

## Conflict of Interest Statement

The authors declare that the research was conducted in the absence of any commercial or financial relationships that could be construed as a potential conflict of interest.
